# Development of Arteannuin B Sustained-Release Microspheres for Anti-Tumor Therapy by Integrated Experimental and Molecular Modeling Approaches

**DOI:** 10.3390/pharmaceutics13081236

**Published:** 2021-08-11

**Authors:** Yanqing Wang, Weijuan Huang, Nannan Wang, Defang Ouyang, Lifeng Xiao, Sirui Zhang, Xiaozheng Ou, Tingsha He, Rongmin Yu, Liyan Song

**Affiliations:** 1 Biotechnological Institute of Chinese Materia Medica, Jinan University, Guangzhou 510632, China; wangyanqing@stu2018.jnu.edu.cn (Y.W.); zsr123@stu2018.jnu.edu.cn (S.Z.); 2 Department of Pharmacology, College of Pharmacy, Jinan University, Guangzhou 510632, China; wjhuang@jnu.edu.cn (W.H.); ouxiaozheng@stu2020.jnu.edu.cn (X.O.); hetingsha@joincare.com (T.H.); 3 State Key Laboratory of Quality Research in Chinese Medicine, Institute of Chinese Medical Sciences (ICMS), University of Macau, Macau, China; mc05838@um.edu.mo (N.W.); defangouyang@um.edu.mo (D.O.); 4 Zhuhai Livzon Microsphere Technology Co., Ltd., Zhuhai 519090, China; xiaolifeng@livzon.cn

**Keywords:** arteannuin B, microsphere, pharmacokinetics, antitumor activity, PLGA, sustained release, molecular modeling

## Abstract

Arteannuin B (AB) has been found to demonstrate obvious anti-tumor activity. However, AB is not available for clinical use due to its very low solubility and very short half-life. This study aimed to develop AB long sustained-release microspheres (ABMs) to improve the feasibility of clinical applications. Firstly, AB-polylactic-co-glycolic acid (PLGA) microspheres were prepared by a single emulsification method. In vitro characterization studies showed that ABMs had a low burst release and stable in vitro release for up to one week. The particle size of microspheres was 69.10 μm (*D*_50_). The drug loading is 37.8%, and the encapsulation rate is 85%. Moreover, molecular dynamics modeling was firstly used to simulate the preparation process of microspheres, which clearly indicated the molecular image of microspheres and provided in-depth insights for understanding several key preparation parameters. Next, in vivo pharmacokinetics (PK) study was carried out to evaluate its sustained release effect in Sprague-Dawley (SD) rats. Subsequently, the methyl thiazolyl tetrazolium (MTT) method with human lung cancer cells (A549) was used to evaluate the in vitro efficacy of ABMs, which showed the *IC*_50_ of ABMs (3.82 μM) to be lower than that of AB (16.03 μM) at day four. Finally, in vivo anti-tumor activity and basic toxicity studies were performed on BALB/c nude mice by subcutaneous injection once a week, four times in total. The relative tumor proliferation rate *T*/*C* of AMBs was lower than 40% and lasted for 21 days after administration. The organ index, organ staining, and tumor cell staining indicated the excellent safety of ABMs than Cis-platinum. In summary, the ABMs were successfully developed and evaluated with a low burst release and a stable release within a week. Molecular dynamics modeling was firstly applied to investigate the molecular mechanism of the microsphere preparation. Moreover, the ABMs possess excellent in vitro and in vivo anti-tumor activity and low toxicity, showing great potential for clinical applications.

## 1. Introduction

**Figure 1:**
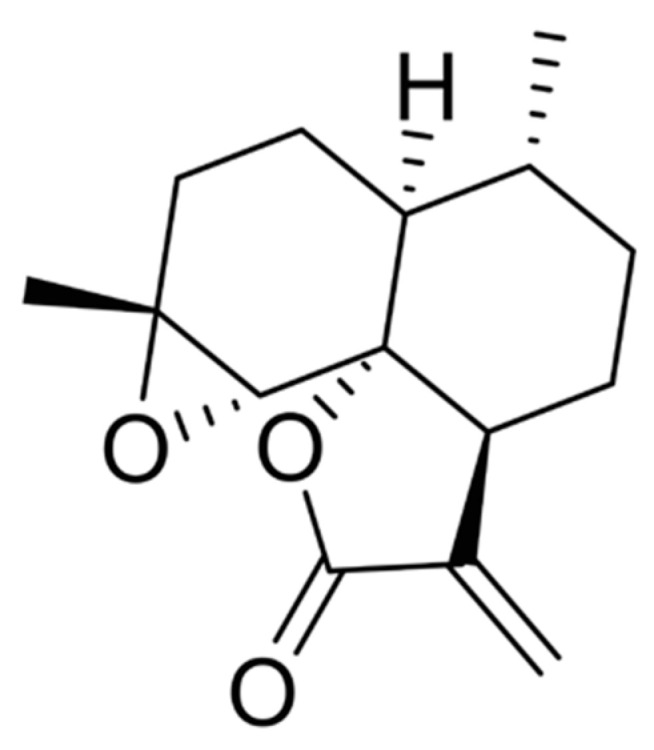
The chemical structure of AB.

Arteannuin B (AB) was first isolated in 1973 from *Artemisia annua* L. and was also a precursor of arteannuin, a sesquiterpene lactone compound with antimalarial effect [1,2]. Compared with traditional arteannuin derivatives, it has no peroxy bridge structure, as shown in Figure 1. Modern pharmacological researches have shown that AB has no antimalarial activity [3], but it exhibits strong anti-inflammatory and immunological activities in preliminary screening [4]. With the deepening of research, people gradually discovered some new pharmacological activities of AB. For example, Qiang et al. [4] found that AB demonstrates significant immunosuppressive activity and also has a certain role in anti-neuroinflammation and treatment of neurodegenerative diseases. Our preliminary experiments found that AB exhibited a good therapeutic effect on mouse lung cancer metastasis models. It can also inhibit the proliferation of HT-29, HepG2, SGC-7901, A549, K562, B16-F10, and Du145 cells, while having no obvious toxicity to normal human embryonic liver cells L-02. Although AB has been known about for over 40 years, there remains very little research on this area, and there are no clinical studies or marketing applications related to AB, which may due to the poor draggability of AB. Firstly, the aqueous insolubility of AB causes its dissolution rate in organisms to be very low, which affects the absorption of the compound, and the bioavailability of AB in animals is only 19–35% [5]. Secondly, the elimination half-life of AB is very short [6], only about 1 h, so it needs to be administered frequently each day, and this will be a great inconvenience to patients [7].

Drug-loaded microspheres are tiny spherical polymers that are made of macromolecules and biodegradable materials with a particle size of a few micrometers to hundreds of micrometers and loaded with one or more drugs [8]. Microspheres could release encapsulated drugs from weeks to months and modify the release profile of drugs [9]. Microsphere formulation has been used for low solubility and low permeability drugs to improve the therapeutic effect and reduce side effects [10,11]. This formulation technology was developed 70 years ago, and the encapsulated drugs are mainly polypeptides [12], proteins [13], and DNAs/RNAs [14,15]. The advantages of microsphere formulation include avoiding the peak and valley phenomenon of the loaded drug in blood concentration, reducing toxic side effects and the dosage in each treatment cycle, and improving the bioavailability of the drug and the compliance of patients. Therefore, microspheres techniques have been widely applied in drugs with a short half-life, medications for chronic diseases, local anesthetics, ophthalmic preparations, anticancer treatment, and have gradually become a popular research direction in modern pharmacy [16]. Currently, there are 13 microsphere products on the market, only three of which are loaded with small molecules, and none of them are anti-tumor drugs. Poor control of drug release rates, inactivation of drugs during fabrication, and difficulties in large-scale manufacturing are recognized as three main disadvantages for microspheres [17]. They were also the practical problems and challenges associated with the application of microspheres. Due to the high toxicity of the anti-tumor drug itself and the high burst release of general microspheres, anti-tumor small-molecule drugs have not been developed as suitable long-acting microsphere preparations for clinical application.

Generally, microspheres can be prepared in several ways, such as emulsion-solvent evaporation, emulsion cross-linking, emulsion-solvent diffusion, coacervation, membrane emulsification, spray drying, and supercritical CO_2_ drying [18,19,20]. However, emulsion cross-linking is not compatible with biodegradable polymers. Spray drying is hard to manage the particle size in a relatively narrow distribution. Membrane emulsification is unable to industrialization although it can provide a very uniform size of particles. Emulsion-solvent diffusion is another common method for generating tidy and neat high-density particles but a very high cost for industrial scale [21]. Benefitting from long-term researches in academia and industry, mature manufacturing technology, batch-to-batch durability, and economical production, the emulsion-solvent evaporation method is more preferred among pharmaceutical companies. In addition, the development of biodegradable polymers also made a great contribution to emulsion-solvent evaporation. For example, PLGA is one of the most promising polymers approved by the Food and Drug Administration (FDA) for human use [19].

The complex pharmaceutical functions of drug products (including the sustained release function of microspheres) are primarily due to beneficial microscopic physicochemical interactions between drugs and excipients, which are difficult to explore by macroscopic experimental tools. Molecular dynamics (MD) simulation is an in silico means of microscopic simulation of the motion state of molecular and atomic systems [22]. In recent years, MD simulations have played a unique role in the field of pharmaceutical science. Pharmaceutical scientists have successfully studied the microstructure and functional mechanisms of drug-delivery systems such as drug-cyclodextrins [23,24], solid dispersions [25], and drug-phospholipid complexes [26] with the help of MD simulations.

In order to further improve the therapeutic potential of AB in clinical applications, this study aimed to develop AB-PLGA sustained-release microspheres using the emulsion-solvent evaporation method. Due to the high dose (~100 mg/day) of AB in the potential therapeutic area, one-week sustained-release microspheres with high drug loading are developed by optimizing a variety of preparation process parameters. These microspheres can release continuously and steadily in the body for one week to improve drug delivery compliance. In vitro and in vivo experiments of ABMs will be compared with AB. Moreover, MD simulations are innovatively introduced to gain some new perspectives to study the microsphere formation mechanism at the atomic level and improve the time efficiency of formulation optimization procedures. Therefore, AB-loaded microsphere formulations may become the first microsphere product of small molecule anti-tumor drugs.

## 2. Materials and Methods

### 2.1. Materials and Reagents

AB was purchased from Nanjing Zhicui Biological Technology Co., Ltd. (Nanjing, China). Poly (lactic-co-glycolic acid) (PLGA), including Resomer^®^ 5050 MN2300, Resomer^®^ 5050 1A, Resomer^®^ 5050 2.5A, Resomer^®^ RG 502H, and Resomer^®^ 7525 5A, were all provided by Evonik Industries (Shanghai, China). Polyvinyl alcohol (PVA) was purchased from Mitsubishi Chemical (Tokyo, Japan). Dichloromethane (DCM) was obtained from Sino Pharm (Beijing, China). Mannitol was purchased from Roquette (Lianyungang, Jiangsu, China). Aripiprazole-D8 solution (100 μg/mL) was obtained from Sigma-Aldrich (Round Rock, TX, USA). Sodium carboxyl methylcellulose (CMC-Na) was purchased from Ashland China (Nanjing, China). Injectable grade Tween 80 was obtained from Nanjing Weier Pharmaceutical (Nanjing, China). Solvents of HPLC grade (acetonitrile, methanol, ethanol, dimethyl sulfoxide (DMSO)) were from Fisher Scientific (Waltham, MA, USA). Isoflurane was from RWD Life Science (Shenzhen, Guangdong, China). Ethanol (75%) was from Realcan (Yantai, Shandong, China). Cis-platinum diamine-dichloride (DDP) injection was kindly gifted from The Fifth Affiliated Hospital of Zunyi Medical College (Zhuhai, Guangdong, China). The solvent for ABMs was prepared by containing 1.125% (*w*/*v*) CMC-Na, 0.8% (*w*/*v*) mannitol, and 0.075% (*w*/*v*) Tween 80 in injectable water.

### 2.2. Preparation of ABMs

ABMs were prepared by the emulsion-solvent evaporation method. In brief, AB (1300 mg), PLGA (1700 mg) were firstly dissolved in DCM (7200 mg) in a dried 25 mL beaker. After dissolution completely, the reaction mixture was allowed to emulsify in 3000 mL (0.5% PVA) aqueous solution at 1800–1900 rpm by a high-shear emulsifier (L5T, Silverson Machines Ltd., Waterside, UK). Next, the emulsion was stirred with a magnetic stirrer (RCT basic, IKA, Staufen, Germany) at room temperature and 800 rpm for 4 h to completely solidify the microspheres, and then the solidified microspheres were filtered with a microporous membrane (1500 mesh, Shanghai Suyan Screen Products Co., Ltd., Shanghai, China) to obtain a crude product of microspheres. Finally, the microspheres were obtained by uniformly re-dispersing in a 10% mannitol aqueous solution and were subsequently freeze-dried (Epsilon 2–6D LSCplus, Christ, Germany).

### 2.3. Drug Loading and Encapsulation Efficiency of ABMs

The microspheres (10 mg) were added to the mixture of acetonitrile and methanol (1:1) and fully shaken to dissolve completely, and then filtered with a 0.22 μm microporous membrane (organic membrane) to obtain the test solution. The peak area was determined by HPLC, and the external reference method was used to calculate the drug loading (*DL*) according to the following equation:

(1) $$DL\;\left(\%\right)=\frac{A_{\left(sample\right)}\times C_{\left(reference\right)}\times D}{A_{\left(reference\right)}\times W}\times 100\%\;$$

where *A*_(*sample*)_ is the peak area of the sample, *A*_(*reference*)_ is the peak area of the reference, *C*_(*reference*)_ is the concentration (mg/mL) of the reference, *D* is the dilution times, and *W* is the weight (mg) of the sample.

Based on the *DL* of ABMs, the encapsulation efficiency (*EE*) is calculated according to the following equation:

(2) $$EE\left(\%\right)\frac{W_{\left(sample\right)}\times DL}{W_{0}}\times 100\%\;$$

where *W*_(*sample*)_ is the total weight (mg) of ABMs, *DL* (%) is the drug loading of ABMs, and *W*_0_ is the weight (mg) of AB used to prepare ABMs.

The detection adopts Agilent 1260 type HPLC, and the detector is Agilent G1314F 1260 VWD detector. The data processing system is an Agilent open LAB chromatography workstation. The chromatographic column is Thermo Syncronis C18 (150 mm × 4.6 mm, 5 µm), the column temperature is 35 °C, the flow rate is 1.0 mL/min; the detection wavelength is 210 nm, the injection volume is 10 μL, and the mobile phase is acetonitrile: water (55:45).

### 2.4. Scanning Electron Microscope

A small amount of ABM powder was adhered to the sample table with conductive adhesive and the unadhered microsphere powder was blown off with compressed air. After using an ion sputtering instrument (LJ-16, Yulong Technology, Beijing, China) to spray gold and make the microspheres conductive, they were placed under a scanning electron microscope (Pro, Phenom World, Eindhoven, The Netherlands) to observe the microscopic morphology.

### 2.5. Characterizations of Particle Size and Particle Size Distribution

About 40 mg ABMs sample was weighed and added to 5 mL ultrapure water. After sonicating for 5 min, the sample was analyzed by a laser particle size analyzer (Mastersizer 2000, Malvern Instruments Ltd., Malvent, UK) to measure their particle size and the width of the particle size distribution. The *D*_10_, *D*_50_, *D*_90_, and *SPAN* of the microspheres were recorded to determine the particle size and distribution. The *SPAN* value of the microspheres can reflect the dispersibility of the particles, and the calculation equation is as follows:

(3) $$SPAN=\frac{D_{90}-D_{10}}{D_{50}}\;$$

where *D*_10_, *D*_50_, and *D*_90_ are the respective diameter of particle size, representing that 10%, 50%, or 90% cumulative volume of the particles are smaller than the specific size.

### 2.6. Molecular Dynamics Simulations of ABMs Preparation Processes

To deeply investigate the mechanism of microspheres formation, MD simulations were used to simulate the preparation processes of ABMs. Before performing the simulation, the simulated system was built. Firstly, the AB molecules, the DCM molecules, and the PLGA fragments with six repeating units were built in BIOVIA Discovery Studio Visualizer. Subsequently, the Antechamber module in the MD simulation software Amber18 (University of California, San Francisco, CA, USA) [27] was used to obtain the missing force field parameters of these structures in the General Amber force field (Gaff) [28] as well as to generate residue topology files. Next, 20 AB molecules, 323 DCM molecules and 4 PLGA fragments in the same proportions as the microsphere preparation were randomly distributed by the Packmol [29] software in cubes with periodic boundaries of 60 Å side length. Finally, the tleap program of Amber18 was used to load the Gaff, which is the force field used in this MD simulation study, and tleap was used to generate the initial coordinate file and topology file for the entire simulated system.

After completing the simulation system preparation, three simulations were performed using the classical MD simulation software Amber18, which was referenced to the single emulsion preparation processes of ABMs. First, AB molecules and PLGA fragments in DCM were subjected to a dynamics simulation lasting 1 ns, which was used to simulate the process of AB dispersed in the oil phase (DCM) with PLGA in the microsphere preparation procedure. The tleap program was then used to add the TIP3P water box to the system at the end of the previous simulation step as the initial structure for the simulation of the O/W emulsification procedure. A 10 Å water layer was set outside the simulated microsphere system for buffering. In this step, the simulation process lasted for 50 ns to ensure the convergence of the simulated system. Finally, the last flame of the previous simulation step was extracted, and the oil phase was removed to be the initial structure for the subsequent MD simulation, which lasted for 100 ns in the periodic boundary TIP3P water box to simulate the oil phase removal and the microspheres curing processes.

Each of the above simulation procedures consisted of four steps. Briefly, first, the solute is fixed, and the solvent is run through a minimization procedure to reduce bad atom contacts. Subsequently, the entire system was also minimized with a 5000-step steep descent minimization followed by 5000 steps of the conjugate gradient minimization. Next, the entire system was heated from 0 K to 300 K in 20,000 steps (40 ps) and held for 60 ps. Finally, the MD simulation was produced, and the trajectory file of the simulation process stored every 10 ps. The whole simulation process can be visualized using Visual Molwcular Dynamics (VMD 1.9.3, University of Illinois at Urbana-Champaign, IL, USA) [30] software. The analysis of the simulation results is done with the help of the CPPTRAJ [31] tool in Amber18.

### 2.7. Characterizations of In Vitro Release

The release medium is 220 mL buffer solution with potassium dihydrogen phosphate (KH_2_PO_4_) and sodium hydroxide (NaOH), both of 0.05 mol/L, Tween 80 in 0.5% (*w*/*v*) in ultrapure water. Then, a pH meter was used to measure and adjust its pH to 7.40.

Microspheres 40 mg were weighed precisely and placed in a 250 mL flask containing 220 mL of release medium and placed in a 37 °C ± 1 incubator (model BF115, BINDER, Tuttlingen, Germany). The solution (1 mL) was taken from the flask with a pipette before and 1, 4, 8, 24, 96, and 168 h after setting out and compensated with 1 mL of the release medium at the same temperature. The extracted solution was filtered through a 0.22 μm microporous membrane (Tianjin Jinteng, Tianjin, China) to obtain the test solution, and then the peak area was measured by HPLC, and the content was calculated by the external reference method. The cumulative release is calculated by the following equation:

(4) $$Cumulative\;Release\;\left(\%\right)=\frac{C_{i}\times V+\sum m_{i-1}}{W_{\left(sample\right)}\times DL}\times 100\%\;$$

where *V* is the volume (mL) of release medium, *∑ m_i_*_−1_ is the total amount of samples taken, *W*_(*sample*)_ is the weight (mg) of ABMs, *DL* (%) is the drug loading, and *C_i_* is the concentration (mg/mL) of test product at the sampling point, as calculated by the following equation:

(5) $$C_{i}=\frac{A_{\left(sample\right)}\times C_{\left(reference\right)}\times D}{A_{\left(reference\right)}}\times 100\%\;$$

where *A*_(*sample*)_ is the peak area of the sample, *A*_(*reference*)_ is the peak area of the reference, *C*_(*reference*)_ is the concentration (mg/mL) of the reference, and *D* is the dilution times.

### 2.8. Cell Culture

The human non-small cell lung cancer (NSCLC) cell line (A549) was from American Type Culture Collection (ATCC, Manassas, VA, USA). RPMI-1640 medium and fetal calf serum (FBS) were from Gibco Life Technologies (Grand Island, NY, USA). A549 cells were inoculated in RPMI 1640 medium (containing 10% FBS) in a 37 °C, 5% CO_2_ cell incubator for culture and storage.

### 2.9. Anti-Tumor Cell Proliferation Activity

A549 cells were seeded into a 96-well plate at a concentration of 5 × 10^3^ per well. AB and ABMs were prepared with DMSO and a specific solvent to make the final concentration per well was 6.25, 12.5, 25, 50, and 100 μM. Here, the dosage of AB in ABMs is calculated according to the actual cumulative release amount in the solution, which is calculated as 55% in 96 h. In the experiment, the control group consisted of cells treated with DMSO and a specific solvent of ABMs and cells without any treatment. The MTT method was used to measure the optical density (*OD*) values of the cells at 24, 48, 72, and 96 h after treatment. The inhibition rate (*IR*) of each group of samples on tumor cell growth was calculated according to the following equation:

(6) $$IR\left(\%\right)=\frac{OD_{\left(solvent\right)}-OD_{\left(exp\right)}}{OD_{\left(solvent\right)}}\times 100\%$$

where *OD*_(*solvent*)_ is the mean *OD* of the solvent group and *OD*_(*exp*)_ is the mean *OD* of the experimental group.

A dose–response curve could be obtained with different concentrations of the samples on the tumor cell growth inhibition, from which the half-inhibition concentration (*IC*_50_) of the sample can be obtained. There are five replicate holes in each group, and the average value of the five holes was taken. Three parallel experiments were carried out for each sample, and the results were expressed as mean ± standard deviation (mean ± SD). According to the inhibition rate of each group, the *IC*_50_ of AB and ABMs at each time point was calculated by the statistical software IBM SPSS Statistics 20.

### 2.10. Animal Treatment

Animal experiments were approved by the Biomedical Ethical Committee of Zhuhai Livzon Microsphere Technology Co. Ltd. (Guangdong, China) (SYXK(Yue) 2016-0158, approval date: 27 July 2016) and were in the light of the Guide for the Care and Use of Laboratory Animals published by the US National Institutes of Health. Efforts were made to reduce the number of animals used and their distress. Male Sprague-Dawley rats (220 ± 20 g) were provided by Guangdong medical laboratory animal center (SCXK(Yue) 2016-0158) (Guangdong, China), and male BALB/c nude mice (22 ± 5 g) were purchased from Beijing HFK Bioscience Co. Ltd. (Beijing, China) (SCXK(Jing) 2019-0008). Throughout the experiments, all the animals had free access to water and food and were housed in an environmentally controlled breeding room (temperature of 20~25 °C, relative humidity of 50~60%) with a 12-h light-dark cycle. Prior to formal experimentation, each animal was housed in a metabolic cage for 5 days to allow for acclimation to the environment.

### 2.11. Administration and Blood Sampling in Rats

There were 9 SD rats equally separated into three groups. The first two groups were injected with AB at a dose of 3.5 mg/kg, through different injection routes, either subcutaneous or intramuscular injections. Collect whole blood from the jugular vein of each rat one day before administration and 0, 0.08, 0.16, 0.25, 0.5, 1, 2, 4, 6, and 24 h after administration. The third group was a subcutaneous injection of ABMs with a dose of 50 mg/kg. The whole blood was collected from the jugular vein of each rat one day before the administration and 0.08, 0.25, 0.5, 1, 2, 4, 6, 24, 72, 168, and 240 h after administration. A 1.0 mL disposable syringe was used, and the blood volume was 0.2 mL each time. The whole blood sample was placed in a centrifuge tube (1.5 mL, Xinkang Medical, Taizhou, Jiang Su, China) containing 2000 KIU aprotinin and EDTA-K_2_ anticoagulant. Turn the centrifuge tube upside down to mix the anticoagulant with the blood thoroughly. Before centrifugation, it should be stored in a pre-cooled CoolRack module (Rack M30, Corning, NY, USA) on wet ice and centrifuged within 15 min after collection. Centrifugation was performed at 4000 rpm, 15 min, 4 °C. After centrifugation, the plasma was separated, the supernatant transferred to the label sample tube (the transfer process needs to be operated on ice), and the sample was checked and stored in the −80 °C refrigerator until analysis.

### 2.12. In Vivo Anti-Tumor Activity

A549 xenograft tumor model was obtained by subcutaneously injecting 1 × 10^6^ cells (A549) into BALB/c nude mice. When the average size of the tumor reached about 100 mm^3^, the tumor-bearing mice were randomly divided into 6 groups (6 mice in each group). The average size of tumors was calculated according to the equation 0.5 × a × b × b (a is the length, b is the width). The 700, 350, and 175 mg/kg dose groups of the ABMs were suspended in a specific solvent and then administered subcutaneously once a week, 4 times in total. The dose of DDP in the positive group was 3 mg/kg, administered once every 3 d until 28 d. The vehicle (microsphere without AB) group was given once a week for a total of 4 times. The model (saline) group was given once a day as a control. Meanwhile, a group of mice without any treatment (WT group) was selected as the standard group for comparison. The changes in food intake, water consumption, and mental state of the nude mice were observed and recorded every day after administration. The body weight and tumor size of the tumor-bearing mice were measured twice a week, and the relative tumor volume (*RTV*) of the nude mice was calculated by Equation (7).

*RTV = V_t_/V_0_* (7)

where *V_t_* is the tumor volume at each measurement and *V*_0_ is the tumor volume before administration. The relative tumor growth rate *T*/*C* (%) was calculated according to the following equation to evaluate the anti-tumor activity of each group.

(8) $$T/C\left(\%\right)=\frac{T_{RTV}}{C_{RTV}}\times 100\%$$

Four weeks after the administration, the nude mice were sacrificed. The heart, liver, kidney, and spleen were dissected, weighed, and recorded, and the viscera index of each organ was calculated according to the following equation:

(9) $$Viscera\;Index\left(\%\right)=\frac{W_{\left(organ\right)}}{W_{\left(body\right)}}\times 100\%$$

The organs and tumors were fixed in 4% formalin, embedded in paraffin, and sections were stained with hematoxylin and eosin (H&E). All sections were observed using an upright white light camera microscope (model Eclipse Ci-L, Nikon, Minato, Tokyo, Japan). In this study, the experimental results of nude mouse body weight, nude mouse tumor volume, relative tumor proliferation rate *T*/*C*, organ index, and tumor quality were all expressed as mean ± standard deviation (mean ± SD).

### 2.13. Analysis of the Concentration of AB in Plasma

LC-MS spectrometry (TQ XS, Waters, Milford, MA, USA) was used to detect the plasma concentration of AB. The electrospray ion source (ESI+) in positive ion mode has an ion source temperature of 150 °C, a solvent removal temperature of 500 °C, and a capillary voltage of 0.5 kV. Multiple reaction monitoring (MRM) mass spectrometry parameters are: AB, *m*/*z* 249.1→189.1, cone voltage 20 V, collision voltage 10 eV; aripiprazole-D8 (internal reference), *m*/*z* 456.3→293.2, the cone voltage is 20 V, and the collision voltage is 25 eV.

SD male rat plasma 50 μL and 200 μL of precipitant (0.1% formic acid in acetonitrile, containing 0.2 ng/mL aripiprazole-D8 as an internal reference) was added into a 96-well plate, and the plate was shaken at 750 rpm (Compact Digital MicroPlate Shaker, Thermo fisher, Waltham, MA, USA) for 10 min. Next, the sample was centrifuged (Legend Micro 21R, Thermo fisher, Waltham, MA, USA) at 4200 rpm for 25 min. After centrifugation, 60 μL of the supernatant was added to a 96-well plate, and then 140 μL of pure water was added, and the plate was shaken at 750 rpm for 5 min before the sample was injected for detection.

HPLC was equipped with a C18 column (Waters, BEH, 130 Å, 2.1 × 50 mm, 1.7 μm), and the mobile phase consisted of elution A (0.1% *w*/*v* citric acid in 5%w acetonitrile aqueous solution) and elution B (0.1% *w*/*v* citric acid in 95%w acetonitrile aqueous solution) at the ratio of 70:30 *v*/*v*. The flow rate was set at 1 mL/min, and the injection volume was 10 μL. The column temperature was set at 60 °C.

### 2.14. Statistical Analysis

All statistical analyses were performed using a two-tailed analysis with a test level set at 5% or *p* ≤ 0.05. The results of all numerical variables were analyzed by statistical mean, standard deviation, and graphical analysis in GraphPad Prism 9.1.2.226 software (San Diego, CA, USA). All data statistical analyses were carried out using IBM SPSS Statistics 20 (IBM, Armonk, NY, USA). First, the numerical variables were tested for Shapiro–Wilk normality and Levene’s test homogeneity. If it was normal (*p* > 0.05) and the homogeneity of variance (*p* > 0.05), then the analysis of variance test was considered. Otherwise, LSD multiple comparisons were performed. If the Shapiro–Wilk results did not conform to normality (*p* ≤ 0.05) and uneven variances (*p* ≤ 0.05), the Kruskal–Wallis nonparametric test was conducted on the raw data. If the Kruskal–Wallis non-parametric test result was significant (*p* ≤ 0.05), further Kruskal–Wallis one-way ANOVA test (K samples) was used for multiple comparisons.

Before analysis, a sphere test (Mauchly’s test of sphericity) should be performed on the correlation between repeated measurement data. Results where *p* > 0.05 indicate that no correlation existed between the repeated measurement data and the measurement data meets the conditions, which can be processed by the one-way analysis of variance method. Otherwise, it indicates a repeated measurement data correlation and the one-way analysis of variance method cannot be processed. At this time, LSD multiple comparisons were performed.

## 3. Results and Discussion

### 3.1. Formulation Screening of ABMs

There are several main evaluation points of the drug-carrying microspheres, the in vitro release of the drug, the apparent morphology, and particle size distribution of the microsphere. The preparation process focuses on screening and optimizing these points, followed by in vivo experiments as further evaluations. Given the aqueous solubility issue of AB, the single-emulsion method was used for the preparation of ABMs. PLGA is the main skeleton material for microspheres. Its physical and chemical properties play a vital role in the morphology of microspheres and the release behavior of loaded drugs [32]. The factors include the molecular weight of PLGA and the ratio of LA to GA, terminal functional groups used, etc. These factors are reflected in the selection of different PLGA models, the ratio of drugs and excipients, and the homogenizer speed in the emulsification process. For this reason, the experiment in this article will use a single variable to carry out the above parameters.

#### 3.1.1. Screening of PLGA

**Figure 2:**
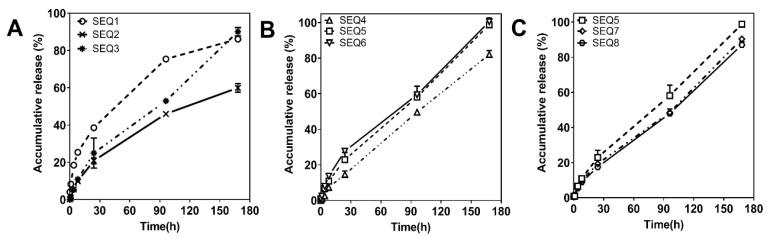
In vitro release profiles of ABMs with different SEQs. (**A**) Different types of PLGAs; (**B**) Different stirring rate of emulsification; (**C**) Different ratios of AB to PLGA (Mean ± SD).

According to the characteristics of PLGA, several clinical approved PLGAs were selected for microsphere preparation experiments: Resomer^®^ 5050 MN2300, 5050 1A, 5050 2.5A, RG 502H, and 7525 5A. These five different types of PLGA microspheres were first prepared as a ratio of AB to PLGA of 1:2, and the concentration of PLGA in the organic solvent was 19% Wt. Scanning electron microscope (SEM) graphs of ABMs prepared by these PLGAs are shown in Supplementary Figure S1. When using 5050 MN2300 to prepare microspheres, the yield of microspheres was low after the solidified liquid filtration, which was not suitable for industrial manufacturing. From SEM, the spherical shape appearance of the microspheres prepared by 5050 1A was irregular. Some of the spheres were damaged, and the microspheres appeared sticky that blocks on the filter. When prepared with 502H, 7525 5A, and 5050 2.5A, the microspheres are complete, uniform, and evenly distributed. Next, the release profiles of AB at different time points (0, 1, 4, 8, 24, 96, and 168 h) of sequence (SEQ) 1, 2, and 3 microspheres in vitro were measured, as shown in Figure 2A. It can be seen from the results that in SEQ 1 the release amount of AB reached up to 38.61% ± 0.90% within 24 h, which was too fast. Although the release amount of SEQ 2 was 20.87% ± 1.60% within 24 h, the accumulative release from 24 to 168 h was relatively slow, which could not meet the requirement of at least 85% drug release within a week.

In order to optimize ABMs, the appropriate types of PLGAs with a suitable loading amount of AB should be considered. When a more lipophilic and high molecular weight 7525 5A was used, the release rate of AB from the microspheres slowed down at 58.30% in 168 h, and the release period was prolonged. When using a low molecular weight 5050 MN2300 (2.3 kDa), the prepared microspheres were soft, and the strength was insufficient after solidification. Hence, using 5050 1A (5 kDa), the strength of the microspheres was significantly improved, but the viscosity may be insufficient, resulting in incompletely wrapped AB within the spheres, and some spheres were damaged during preparation. RG 502 H has a slightly higher molecular weight (7–17 kDa, 50:50), which solved the problem of sphere integrity, but there was an obvious burst and excessive release. It was necessary to choose a higher molecular weight 5050 2.5A (20 kDa) to delay the degradation rate of PLGA. In this case, a higher molecular weight PLGA was preferred to generate a good shape of microsphere, allowing good encapsulation rate and low burst release [33] Moreover, the proper ratio of lactic and glycolic acid forming a suitable intermolecular interaction between AB and adjuvant influenced the release profile of ABMs [34]. Therefore, the formulation of SEQ 3 was selected for the next step.

#### 3.1.2. The Stirring Rate of Emulsification

The emulsification step is one of the most critical procedure to prepare different shapes of microspheres [35]. The effects of different ratios of AB to PLGA and different homogenizer speeds in the emulsification process were assessed to optimize the in vitro release profile. The cumulative release of ABMs at different time points was shown in Figure 2B. The higher the stirring rate of emulsification (1900 rpm), the faster the release of the microspheres (100.53% ± 2.15%) within 168 h. The release rate of SEQ 6 82.12% ± 2.23% was slower at 1700 rpm, so that the release rate (98.78% ± 0.28%) at 1800 rpm was the most appropriate. If the stirring rate was more than 1900 rpm, the release rate was too fast, so 1800–1900 rpm was preferred. The possibility to explain the stirring rate affects the release rate was the generation of microspheres with different sizes. When the stirring rate was faster, the particle size of microspheres was smaller, while the tiny particle size would cause a faster release rate.

#### 3.1.3. The Ratio between AB and PLGA

At the same time, increasing the ratio of AB to PLGA, the release rate of microspheres within 168 h was increased accordingly (Figure 2C). The release rate at each time point was slightly slower at SEQ 7 and 8 when the ratio is 1:1 and 1:0.7, respectively. The overall release rate was more suitable at the ratio of 1:1.3. Therefore, the condition of SEQ 5 was preferred. The optimal formulation of SEQ 5 adopted a 1:1.3 ratio of AB and PLGA (5050 2.5A) and 1800–1900 rpm as emulsification stirring rate.

### 3.2. Characterization of ABMs

The size, apparent morphology, and *EE* are often considered as the critical quality attributes [32]. In addition, pH and osmotic pressure are demonstrated ass remarkable influences on the safety of injections.

**Figure 3:**
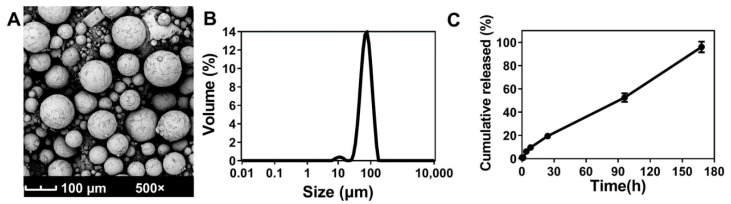
Characterizations of ABMs. (**A**) Image of ABMs; (**B**) Partial size distribution of ABMs; (**C**) In vitro release of ABM (Mean ± SD).

With a high drug loading (37.8%) and encapsulation efficiency (85%), further characterizations of SEQ 5 were performed. Osmotic pressure was 308 mmosm/kg, pH value was 6.2, particle size *D*_10_ (41.41 μm), *D*_50_ (69.10 μm), *D*_90_ (110.31 μm), and *SPAN* was 0.997. These parameters were met with the requirements of China Food and Drug Administration [36]. Porous microspheres with smaller particle size exhibited faster release kinetics for drug diffusion due to the enhancement of surface area [37,38,39]. From the SEM micrograph (Figure 3A) and the width of the particle size distribution diagram (Figure 3B), although a small portion of about 10 μm size microspheres was detected, limited influence on the burst effect about 10% (0–8 h) was observed. According to the release profile of ABMs, the cumulative release (0–168 h) was over 98%, which was close to complete release, and the release rate after 8 h was basically constant and linear (Figure 3C).

### 3.3. Molecular Dynamics Simulations of ABMs

**Figure 4:**
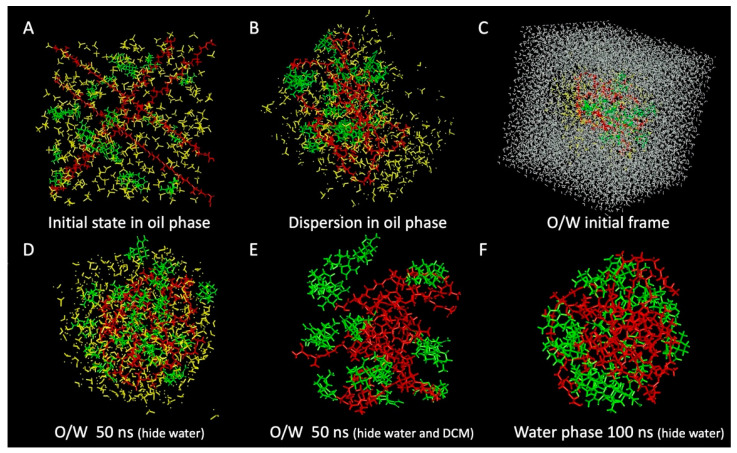
Snapshots of MD simulations of ABMs in the oil phase at (**A**) 0 and (**B**) 1 ns, in the O/W system at (**C**) 0 and (**D**,**E**) 50 ns, in the water phase at (**F**) 100 ns. Green molecules: AB; red molecules: PLGA; yellow molecules: DCM; gray molecules: water.

As shown in Figure 4, the visualization results of MD simulations show us the intuitive microscopic changes of the microsphere preparation processes. The initial state of the oil phase simulation is shown in Figure 4A. Figure 4B shows that the AB molecules and PLGA molecules are fully dispersed in the oil phase, showing no apparent intermolecular interactions. With the addition of the aqueous phase (Figure 4C), the surface tension between the oil and the aqueous phases causes the molecules in the oil phase to start to aggregate, and the whole oil phase system is sphere-like, as shown in Figure 4D. After hiding the DCM molecules in Figure 4E, we can see that the AB molecules and PLGA molecules still appear loose from each other. Further simulation results of the AB-PLGA system in the aqueous phase are shown in Figure 4F. After the removal of the oil phase solvent, the AB molecules and PLGA molecules are tightly aggregated into a spherical shape in the aqueous phase.

**Figure 5:**
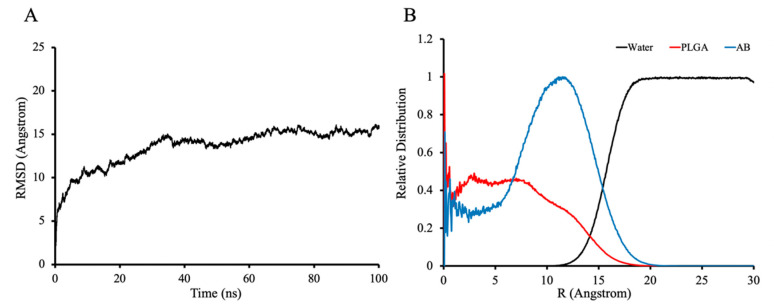
*CPPTRAJ* analysis results of ABMs in water phase simulation. (**A**) Root-mean-square deviation of AB-PLGA. (**B**) Relative distribution of AB, PLGA and water, converted from RDF (radial distribution function) by setting the maximum distribution value to 1.

Figure 5A shows the RMSD (Root-mean-square deviation) curves of the simulation of AB-PLGA in the aqueous phase after the removal of DCM, which indicates that the simulated system converges at about 40 ns. Figure 5B shows the radial distribution of AB molecules, PLGA fragments, and water molecules in the aqueous phase simulation. The horizontal coordinate is the distance from the spherical center of the AB-PLGA system, and the vertical coordinate is the relative distribution, which was converted from RDF (radial distribution function). The maximum distribution in the range of this coordinate system is set to 1 to obtain the relative distribution value. Therefore, the closer the vertical coordinate value is to 1, the denser the distribution of molecules is at that position. Comparing the distribution of AB-PLGA and water molecules, we can see that the hydrophobic AB and PLGA molecules rely on hydrophobic interactions to be tightly bound, and water molecules are almost not distributed inside the microspheres. Moreover, we can see that, in the spherical system, the AB molecules show a staggered distribution with PLGA instead of AB molecules aggregating alone, which is consistent with the stable and sustained release of drug molecules from the PLGA skeleton [40]. In addition, the drug molecules possess a relatively higher distribution around the surface of the sphere, which may also coincide with the initial burst release of drugs around the surface of microspheres [40,41].

In short, the MD simulations successfully simulated the dispersion of drug molecules and PLGA polymer in the oil phase, O/W emulsification process, oil phase removal, and microsphere solidification during the preparation of ABMs. Moreover, the formation mechanism of ABMs was revealed from a microscopic point of view.

### 3.4. Mathematical Model Fitting of ABMs In Vitro Release Mechanism

**Figure 6:**
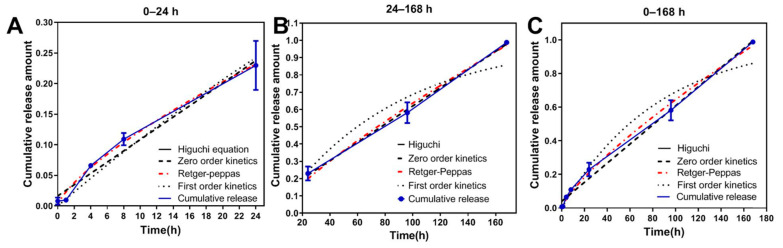
Results of curve fitting of in vitro release of ABMs in different time periods: (**A**) 0–24 h; (**B**) 24–168 h; (**C**) 0–168 h. All numerical results were denoted by mean and SD.

Five release profile equations were selected to perform the in vitro release kinetic model of ABMs: zero-order kinetic equation [42], first-order kinetic equation, Higuchi plane diffusion equation, Hixcon–Crowell corrosion equation, and Ritger–Peppas equation [43,44]. Graphpad Prism 6.0 software was used to analyze line curve fitting and to judge the curve fitting condition according to the goodness of fit. The in vitro release profile of ABMs and the fitting results are shown in Table 1 and Figure 6.

As a result, the in vitro release of ABMs at 0–24 h was best matched the Retger-peppas equation (R^2^ = 0.9915), while 24–168 h fitted with the zero-order release equation (R^2^ = 0.9983) best. Although the result of one week fitted the Ritger–Peppas equation (R^2^ = 0.9959), it was also very close to the zero-order release equation (R^2^ = 0.9929). So, ABMs can release at an approximately constant rate throughout the lifetime. At equilibrium, it was cleared out of the body, and steady drug plasma concentrations were achieved within the therapeutic window without frequent re-dosing [45,46]. Crucially, compared to first-order release and immediate-release systems, the total cumulative dose in the body was also reduced, which ultimately reduced the chronic toxicity risk [42].

### 3.5. PK Study of ABMs

**Figure 7:**
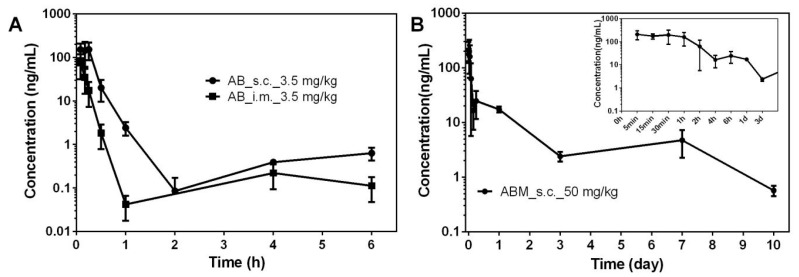
AB blood concentrations in male SD rats. (**A**) Comparison of PK results after intramuscular injection (IM) and subcutaneous injection (SC) of AB; (**B**) PK results of ABMs. All numerical results were denoted by mean and SD.

As there was no research report on the PK of AB in vivo so far, it was necessary to determine the appropriate administration pathway and dosage of ABMs from the PK results of AB. The administration pathway was determined by comparing the difference between subcutaneous and intramuscular injection of AB. The dose of AB is 3.5 mg/kg, and the blood concentration changes at each time point are shown in Figure 7A and the PK parameters are shown in Table 2. The elimination half-life of AB in rats was very short, about 1 h. Therefore, it was not suitable for conventional oral or injection pathways. The generation of sustainable long-acting injections can effectively overcome the problem of short half-life. In addition, AUC_last_ from subcutaneous injection was about four times that of intramuscular injection, and the bioavailability of subcutaneous injection was higher. The rate and extent of intramuscular and subcutaneous absorption were variable based on physical and chemical properties of compounds. The lipophilicity of AB and the difference of blood flow at the injection site played an important role [47,48]. As a result, ABMs were more suitable for subcutaneous administration. ABMs were administered subcutaneously in SD rats at a dosage of 50 mg/kg. The blood sample was collected to the tenth day after administration. The PK curve is shown in Figure 7B and the PK parameters are shown in Table 2.

ABMs have a relatively large release in the first hour, and then the compound could maintain a constant release for a week. The blood concentration on the tenth day decreased significantly, so this formulation was suitable for the one-week sustained release of AB, while AB direct injection required administration multiple times a day to maintain the necessary blood concentration.

### 3.6. In Vitro Anti-Tumor Activity in Cell Experiments

From the results of the *OD* value at each time point, the *IR* of each group on the cells was calculated according to Equation (6). Then, the inhibition rate was used to calculate the *IC*_50_ values of ABMs and AB on cell inhibition at different time points, as shown in Table 3.

There was no significant difference in *IC*_50_ between the AB group and the ABMs group within 24 h after administration, and even the ABMs group was higher than the AB group. This situation may be due to the low release rate of microspheres within 24 h, in which the exposure amount of AB from the microspheres was much lower than AB group. Until the release up to 48 h, the *IC*_50_ value of the ABMs group began to be lower than that of the AB group, and the *IC*_50_ gap between them was getting more prominent as time went on. *IC*_50_ of ABMs group in 96 h was about 1/4 of AB group, indicating that the compound in the microspheres was gradually and slowly released over time, so the anti-tumor cell activity of ABMs gradually increased. In other words, the ABMs displayed cytotoxic potency to the A549 cells proliferation in a time-dependent manner, as a hint that drug diffusion and polymer degradation might be favorable to the drug delivery with the prolongation of incubation [49]. Hence, the anti-tumour efficiency of ABMs was significantly enhanced compared to AB.

### 3.7. In Vivo Anti-Tumor Activity

The in vivo PK results proved that the ABMs could be released continuously for one week. To further verify the rationality of its once-a-week dosing, a study was conducted from the perspective of drug efficacy to investigate the anti-tumor activity of ABMs.

**Figure 8:**
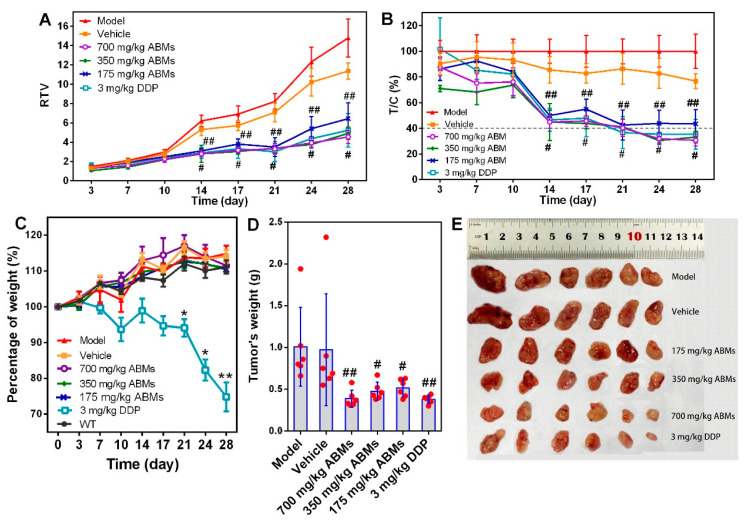
Diagrams of in vivo pharmacodynamics. (**A**) Results of relative tumor volume change over time in tumor-bearing mice within 28 days; (**B**) T/C (%) change result of tumor-bearing mice relative tumor growth rate within 28 days; (**C**) Results of body weight changes over time in different tumor-bearing mouse groups; (**D**) Tumor weights of different tumor-bearing mice groups; (**E**) Diagram of tumor size at the site of inoculation after the experiment in different tumor-bearing mouse groups (*n* = 6). Note: A&B, ^#^ *p* < 0.05, vs. vehicle group; ^##^ *p* ≤ 0.001, vs. Model group; * *p* < 0.05, vs. WT group; ** *p* < 0.01, vs. WT group. All numerical results were denoted by mean and SD.

After the tumor-bearing mice were successfully modeled, they were administered according to the preset dosage and administration method. After administration, the tumor size of each tumor-bearing mice was measured with a vernier caliper twice a week, and the change in relative tumor volume (Figure 8A) and the relative tumor proliferation rate at each time point (Figure 8B) were calculated. Four weeks after the administration, the nude mice were sacrificed, the tumor was dissected, and its weight was recorded (Figure 8C). By the way, the experimental plan is to conduct a comparative study on the efficacy of AB. Because AB is insoluble in water, clinically acceptable Tween-80, ethanol, and PEG-40 were selected for dissolution, but the rats showed significant intolerance symptoms after administration. Therefore, the research on the efficacy of AB was abandoned.

After different doses of ABMs and DDP were administered to tumor-bearing mice from the 14th day, there were no significant statistical differences in *RTV* and *T*/*C* between the WT group and vehicle group, and no significant difference between the ABMs and DDP groups in different doses. Although the *T*/*C* results of 175 mg/kg ABMs were significantly different from the model control group, the *T*/*C* was higher than those of 40% at all time points. It is believed that the effect is not as expected at low doses. On the 24th to 28th day after administration, the *T*/*C* of the 350 mg/kg ABMs and 700 mg/kg ABMs and the DDP group were less than 40% and were significantly different from the model group (*p* < 0.001), indicating that the drug at this dose has a significant inhibitory effect of tumor growth. From the tumor dissected graph and tumor volume graph, each treating group of AB has a certain activity of inhibiting tumor growth, and the tumor size of the 700 mg/kg ABMs group (0.39 ± 0.09 g) is basically the same as the DDP group (0.38 ± 0.04 g).

After the administration, the mental state of each group of nude mice was observed every day, and the body weight was measured twice a week. It was found that the mental state of the nude mice in the positive DDP group was becoming increasingly worse, and their body weight showed a significant downward trend. Their average body weight at 28 days was only 74.8% of that before administration. However, the body weight of other groups increased gradually as the WT group. The average body weight of the high-dose ABMs group and the vehicle group were 111.4% and 114.1% of before administration, respectively, which was consistent with the changing trend of the WT group (increased to 111.1% before administration), as shown in Figure 8C. The stable body weight suggested that the AB-loaded microspheres caused negligible side effects and were suitable for developing for potential clinical treatment. The body weight of the animals in the DDP group decreased significantly from the 10th day and continued to decline to the 28th day, and there was no significant difference between the other groups, as shown in Figure 8C. At the same time, the tumor weight among the various groups tends to decrease with the increase of the dose, but there is no statistical difference (Figure 8D,E). These studies presume that the safety of ABMs is better than the DDP group, because the efficacy of ABMs in the middle and high-dose groups is equivalent to the DDP group. The ABMs possessed superior anti-tumor efficacy that might be attributed to the efficient drug delivery, penetration, and accumulation in tumor sites [49].

**Figure 9:**
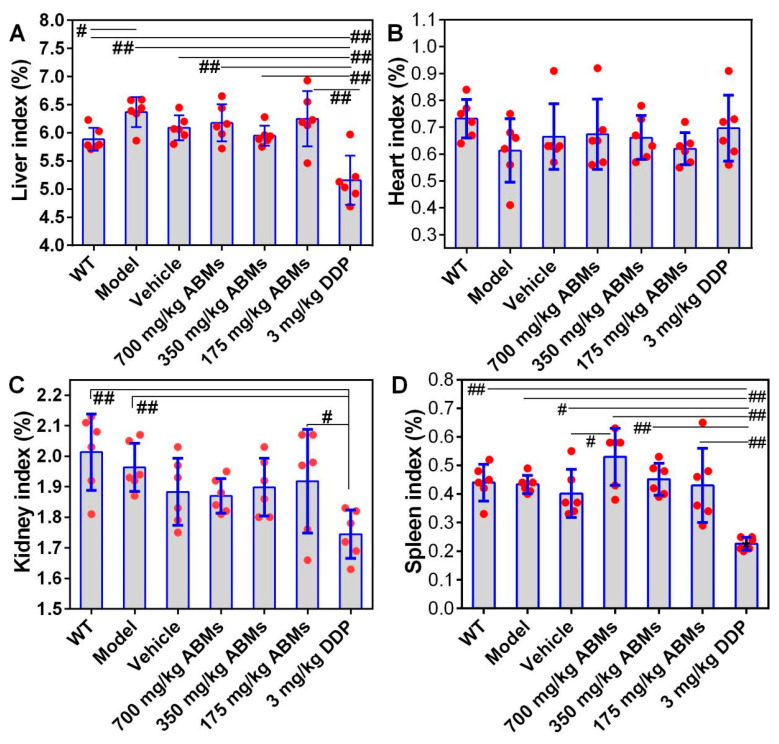
Statistical analysis of organ indexes of tumor-bearing mouses. (**A**) liver; (**B**) heart; (**C**) kidney; (**D**) spleen. Note: ^#^ *p* < 0.05, ^##^ *p* ≤ 0.01. All numerical results were denoted by mean and SD.

After the administration, the nude mice were sacrificed, the heart, liver, spleen, and kidney were dissected and weighed, and the organ index of each group was calculated, as shown in Figure 9. The indexes of the liver (Figure 9A), heart (Figure 9B), kidney (Figure 9C), and spleen (Figure 9D) of the low, medium, and high dose ABMs groups were not significantly different from those of the WT group (*p* > 0.05). The liver index between the model group and the WT group was statistically different (*p* < 0.05). The spleen index of the high-dose ABMs group and the vehicle group was statistically different (*p* < 0.05). However, it is worth mentioning that the organ index between the DDP group and the other groups are significantly different (*p* < 0.001), which further suggests that the different doses of the ABMs group maintain the sustained release of the drug at the same time. Compared with the DDP group, the ABMs groups can greatly reduce security risks. DDP group showed severe spleen toxicity and slight renal toxicity. The spleen showed obvious atrophy, and the spleen index was about half of the normal group (4.39 mg/g in the model group and 2.25 mg/g in the DDP group), and the renal index decreased slightly (20.12 mg/g in the vehicle group and 17.43 mg/g in the DDP group). However, there were no significant changes in the organ indexes of the ABMs in each dose group, the vehicle group, and the model group, indicating that the ABMs and their PLGA have high safety.

### 3.8. In Vivo Toxicity to Various Organs and Tumors

**Figure 10:**
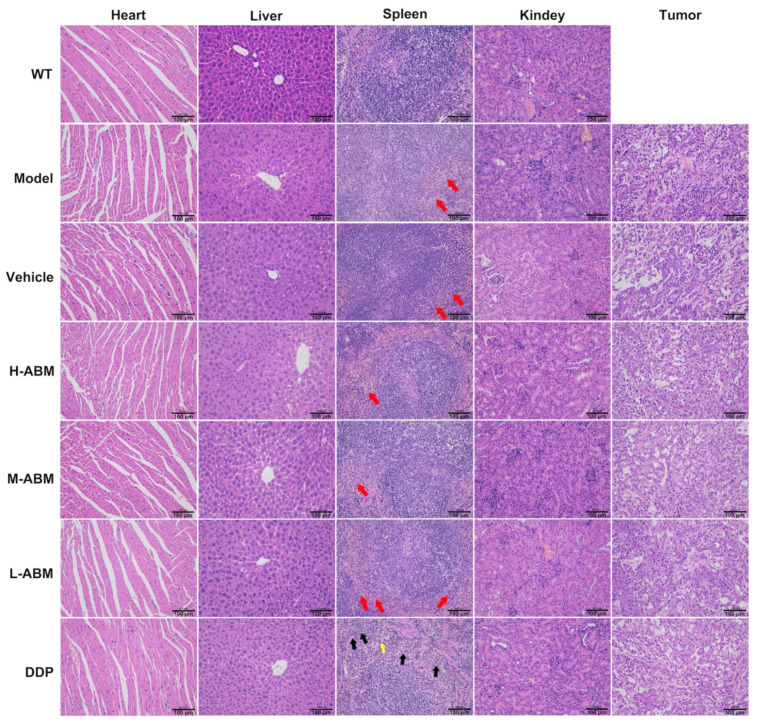
Histological observation of various tissues including heart, liver, spleen, kidney and tumor after hematoxylin and eosin (H&E) staining (Scale bars, 50 μm).

Organs and tumors in each group were stained with hematoxylin and eosin (H&E), as shown in Figure 10. Except for the spleen, no obvious lesions were found in the pathological sections of other organs in each group. In the pathological section of the spleen, the red and white pulp of the spleen in the DDP group was clearly distinguished. Compared with the normal group, the red blood cells in the red pulp decreased, the lymphocyte content increased (black arrows), and more brownish-yellow pigmentation (yellow arrow) was seen [49]. Other than that, no obvious pathological changes in cellular morphology. However, each dose group of ABMs is similar to the model group. The red and white pulp is clearly demarcated. The white pulp is visible in the central artery, lymphocytes are abundant, the morphology is normal, and a small number of scattered neutrophils (red arrows) are seen in the red pulp. No other obvious differences were seen. Consequently, the novel ABMs delivery system contributes to high anti-tumor efficiency and low incidence of side effects observed.

## 4. Conclusions

In summary, the AB-loaded one-week sustained-release microspheres were successfully prepared, characterized, and evaluated in vitro and in vivo, demonstrating a significantly improved, short half-life and efficacy as an anti-tumor drug. Molecular modeling was used the first time to simulate the microsphere preparation processes, which provided the molecular image of microspheres and in-depth insights for further understanding the critical preparation parameters. The AB-loaded microspheres have demonstrated the inhibition of tumor growth, low toxicity, and less dosing frequency, showing great potential for further application in the therapeutic area. Our future research will focus on the dose adjustment for longer release behavior of microspheres in clinical studies.

## Figures and Tables

**Table 1:** Mathematical models fitting results of ABMs release.

Models	Formulas	0–24 h		24–168 h		0–168 h	
		Fitting Equation Results	R^2^	Fitting Equation Results	R^2^	Fitting Equation Results	R^2^
Zero-order kinetic	Q = Kt + b	Q = 0.009220 × t + 0.01629	0.9726	Q = 0.005265 × t + 0.09424	0.9983	Q = 0.005676 × t + 0.04047	0.9929
First-order kinetic	ln(1−Q) = −Kt	ln(1−Q) = −0.01156 × t	0.9672	ln(1−Q) = −0.01161 × t	0.9122	ln(1−Q) = −0.01172 × t	0.9679
Higuchi	Q = Kt^1/2^	Q = 0.04259 × t^1/2^	0.9375	Q = 0.06814 × t^1/2^	0.8985	Q = 0.06670 × t^1/2^	0.9461
Hixcon-Crowell	(100−Q)^1/3^ = −Kt	(100−Q)^1/3^ = 0.1958 × t	−116,8000	(100−Q)^1/3^ = 0.02885 × t	−56,346	(100−Q)^1/3^ = 0.02885 × t	−68,825
Retger-Peppas	Q = Kt^n^	Q = 0.02285 × t^0.7288^	0.9915	Q = 0.01490 × t^0.8154^	0.9917	Q = 0.01763 × t^0.7815^	0.9959

**Table 2:** Pharmacokinetic data of AB and ABMs (Values represent mean ± SEM, *n* = 6).

Sample	Pathway	Dose	t_1/2_	C_max_	AUC_last_
		(mg/kg)	(day)	(ng/mL)	(day·ng/mL)
ABMs	SC	50	2.820 ± 0.350	262.000 ± 146.860	74.774 ± 5.441
AB	SC	3.5	0.048 ± 0.003	235.667 ± 100.431	2.538 ± 0.842
	IM	3.5	0.096 ± 0.052	74.500 ± 34.155	0.618 ± 0.131

**Table 3:** *IC*_50_ values (μM) of AB and ABMs (Values represent mean ± SD, *n* = 3).

Time (h)	24	48	72	96
AB	211.37 ± 63.17	53.10 ± 22.52	27.52 ± 6.71	16.17 ± 4.78
ABMs	290.59 ± 40.06	38.40 ± 9.58	11.13 ± 10.00	3.94 ± 1.80

## Data Availability

Experimental data may be available upon request.

## Supplementary Materials

The following are available online at https://www.mdpi.com/article/10.3390/pharmaceutics13081236/s1, Figure S1: Electron micrographs of ABMs prepared with different PLGAs. SEM images: (A) 5050 1A; (B) 502H; (C) 7525 5A; (D) 5050 2.5A.
