# Supplementary material for: Development of Arteannuin B Sustained-Release Microspheres for Anti-Tumor Therapy by Integrated Experimental and Molecular Modeling Approaches
Source: Pharmaceutics. 2021 Aug 11;13(8):1236. doi: 10.3390/pharmaceutics13081236 (PMC8399913; doi:10.3390/pharmaceutics13081236)
Supplement: Supplementary file 1 [file pharmaceutics-13-01236-s001.zip › pharmaceutics-1300637-supplementary.pdf]

pharmaceutics

MDPI

Supplementary Materials: Development of Arteannuin B Sustained-Release Microspheres for Anti-Tumor Therapy by Integrated Experimental and Molecular Modeling Approaches

Yanqing Wang, Weijuan Huang, Nannan Wang, Defang Ouyang, Lifeng Xiao, Sirui Zhang, Xiaozheng Ou, Tingsha He, Rongmin Yu and Liyan Song

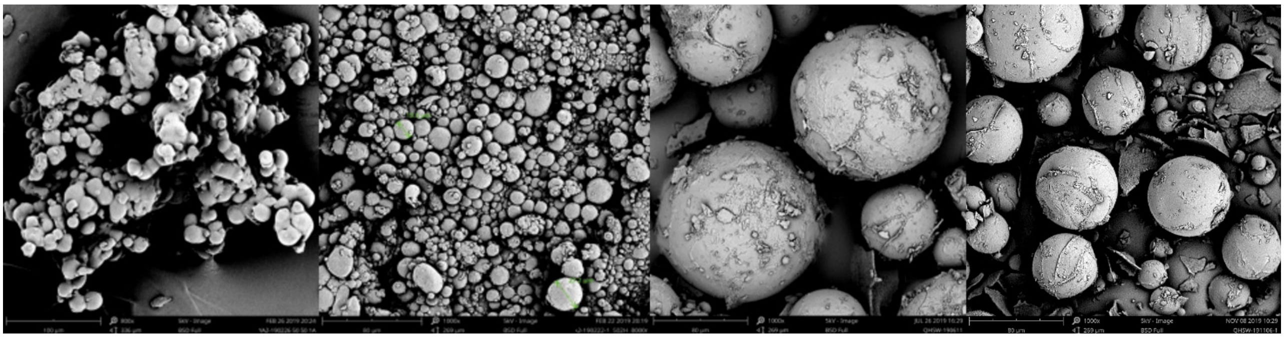

Figure S1. Electron micrographs of ABMs prepared with different PLGAs. SEM images: (A) 5050 1A; (B) 502H; (C) 7525 5A; (D) 5050 2.5A.
